# 400 °C Sensor Based on Ni/4H-SiC Schottky Diode for Reliable Temperature Monitoring in Industrial Environments

**DOI:** 10.3390/s19102384

**Published:** 2019-05-24

**Authors:** Florin Draghici, Gheorghe Brezeanu, Gheorghe Pristavu, Razvan Pascu, Marian Badila, Adriana Pribeanu, Emilian Ceuca

**Affiliations:** 1 Electronic Devices, Circuits and Architectures Department, University Politehnica of Bucharest, Bucharest 060042, Romania; gheorghe.pristavu@upb.ro (G.P.); marian.badila1@yahoo.com (M.B.); 2 National Institute for Research and Development in Microtechnologies, Bucharest 077190, Romania; razvan.pascu@imt.ro; 3 CEPROCIM S.A., Bucharest 062203, Romania; adriana.pribeanu@ceprocim.ro; 4 Exact and Engineering Sciences Department, University 1 Decembrie 1918 of Alba Iulia, Alba Iulia 510009, Romania; eceuca@uab.ro

**Keywords:** high-temperature sensor, read-out circuit, silicon carbide, Schottky diode, harsh environment, industrial temperature monitoring

## Abstract

This paper presents a high-temperature probe suitable for operating in harsh industrial applications as a reliable alternative to low-lifespan conventional solutions, such as thermocouples. The temperature sensing element is a Schottky diode fabricated on 4H-SiC wafers, with Ni as the Schottky metal, which allows operation at temperatures up to 400 °C, with sensitivities over 2 mV/°C and excellent linearity (R^2^ > 99.99%). The temperature probe also includes dedicated circuitry for signal acquisition and conversion to the 4 mA–20 mA industrial standard output signal. This read-out circuit can be calibrated for linear response over a tunable temperature detection range. The entire system is designed for full electrical and mechanical compatibility with existing conventional probe casings, allowing for seamless implementation in a factory’s sensor network. Such sensors are tested alongside standard thermocouples, with matching temperature monitoring results, over several months, in real working conditions (a cement factory), up to 400 °C.

## 1. Introduction

Temperature sensors are required in virtually all monitoring applications, irrespective of investigated settings [1]. As advancements in regulations targeting the minimization of industrial impact on the environment are carried out, precise temperature control and energy efficiency have become essential research topics, which exert ever-growing standards from temperature sensors [1,2,3,4,5,6,7,8,9,10,11,12,13,14,15,16,17,18,19]. This aspect works in tandem with the fact that most industrial processes demand careful monitoring of temperature levels in order to ensure optimal quality and yield [1,6]. Therefore, accurate, robust, and reliable temperature sensors are among the primary requirements in the industrial sector, especially for the cement, drilling, aviation, automotive, or geothermal industries [6]. To cater to these needs, industrial temperature probes need a read-out circuit in addition to the sensing element, in order to adequately acquire and convert its output signal to standard levels [16].

Currently, thermocouples represent the preferred solution for temperature sensing, because of their wide measurement range, good accuracy and fast response time. Industrial working conditions often include elevated heat levels, strong vibrations, corrosion, erosion (etc.), which exact a heavy toll on the reliability of thermocouples, restricting their lifespan [6]. Additionally, a thermocouple’s temperature response is non-linear, with sensitivities usually restricted under 0.1 mV/°C, which considerably increases the expected performances of read-out circuits [17].

As such, wide band-gap semiconductor-based temperature sensors have become a promising alternative for harsh industrial applications [2,3,4,5,6,7,8,9,10,11,12,13,14,15]. Silicon carbide (SiC) is a particularly suitable material due to its mechanical robustness, radiation, chemical inertness, and high bandgap (E_g_ = 3.25 eV for 4H-SiC), which allows fabricated devices to operate at temperatures up to 400 °C or more [2,3,4,5,6,7,8,9,10,11,12,13,14,15]. Among all devices fabricated on SiC (PN, PIN, JFET, etc.), Schottky diodes are the most cost-effective and technologically mature, with considerable commercial success in power and sensing applications [2,3,4,5,6,7,8,9,10,11,12,13,14,15,16,17,18,19,20,21,22,23,24,25,26,27]. Hence, due to their simple structure, very compact size and quasi-linear voltage-temperature dependence (with sensitivities exceeding 2 mV/°C), SiC Schottky diodes are excellent candidates for high-temperature monitoring in hostile industrial environments [2,3,4,5,6,7,8,9,10,11,12,13,14,15,16,18,19,20,21,22,23,24].

This paper demonstrates the viability of SiC-Schottky diode-based high-temperature sensors in industrial applications, which are fully electrically and mechanically compatible with existing probes. Diodes with annealed Ni/4H-SiC contacts, used as sensing element, are designed, fabricated and measured up to 400 °C. Parameter extraction and subsequent modeling of forward behavior evince best performing devices in terms of sensitivity value and stability.

To process the Schottky diode signal, a dedicated read-out circuit was designed and implemented. Thus, sensor output voltage was converted to current in the industrial standard (4 mA–20 mA). Following calibration, the sensor system was assembled and incorporated into an industrial casing. Several such temperature probes were tested in the raw meal mill of a cement factory alongside standard thermocouple-based counterparts for several months. Results showed good agreement between both temperature-sensing solutions and emphasized the exemplary reliability potential of SiC-based devices working in harsh industrial environments.

## 2. Materials and Methods

The proposed sensing element of the temperature probe is a SiC-Schottky diode, due to its ability to operate at elevated temperatures (at least 400 °C). Temperature detection is quantified by exploiting the forward characteristics (I_SD_-V_SD_) of this device. Explicitly, biasing the diode at constant currents leads to a quasi-linear decrease in voltage as temperature rises. The sensitivity is inversely proportional to the current level [6,7,8,9,10,11].

### 2.1. SiC-Schottky Diode Sensor Technology

**Figure 1:**
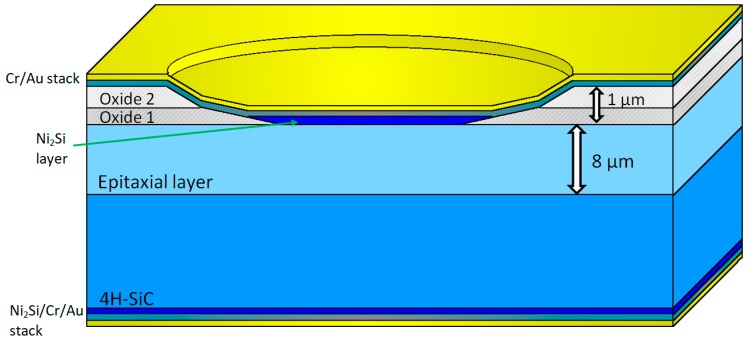
SiC-Schottky diode configuration.

The high-temperature sensors were fabricated on research grade *n*-4H-SiC wafers from Cree Inc. (Durham, NC, USA), (3.85° orientation, Si-face, 20 mΩ/cm resistivity), with an epitaxial layer with a thickness of approximately 8 µm and a concentration of around 1 × 10^16^ cm^−3^. A cross section of this SiC-Schottky structure is depicted in Figure 1. The first technological step was a chemical cleaning process, based on piranha and 10% HF solutions, for 30 s. Then, a two-stage oxide deposition by Low-Pressure Chemical Vapor Deposition (LPCVD) was performed.

An initial oxide layer (Oxide 1—Figure 1) was deposited at a temperature of 750 °C, followed by a densification process at 1000 °C in inert atmosphere (N_2_) for 30 min. The value of the refraction index for as-deposited SiO_2_ was 1.4405 at a wavelength of 632.8 nm, and 1.4392 after the densification, at the same wavelength.

For the ohmic contact, a Ni (150 nm) deposition on the backside of the wafer (with a covered front side) was also performed by sputtering, followed by a rapid thermal annealing at a temperature of 950 °C, for 5 min, in an N_2_ atmosphere.

Afterwards, a second oxide layer (Oxide 2—Figure 1) was added on the front side, over the densified oxide (Oxide 1—Figure 1), leading to a total thickness of around 1 µm.

**Figure 2:**
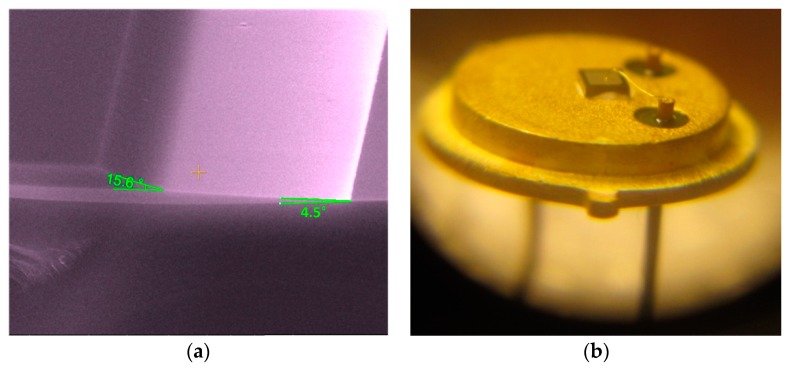
(**a**) Ramp oxide termination profile of SiC-Schottky sensor and (**b**) SiC-Schottky structure on TO39 case (with silver nano-paste).

To ensure a uniform current density, wet etching of the oxide layers, using a photoresist mask, was performed, yielding an oxide ramp termination [6,7,8,9,10,11,12,13,14]. This etching process occurred at a rate of approximately 145 nm/min, in a (NH_4_F/CH_3_-COOH) (2:1) solution. Two different angles (under 20°) were obtained, due to the different densities of the oxide layers, as evinced in Figure 2a. These low angles can be modified by changing the temperature of the etching solution between 25 °C and 40 °C [24]. Hence, circular active windows, with diameters of 400 µm were opened in the oxide layers. Afterwards, 150 nm of Ni were deposited by sputtering on the front side of the wafers for the Schottky contacts. A rapid thermal annealing in the N_2_ atmosphere, at a temperature of 800 °C for 8 min was carried out in order to obtain a Ni-silicide (Ni_2_Si) Schottky contact [6,7,8].

Finally, metallic layer stacks consisting of Cr (20 nm)/Au (200 nm) were deposited on both sides of the wafers in order to achieve the pad contacts (Figure 1). The Cr layer serves to improve the Schottky barrier stability, as it prevents Au diffusion at high temperatures (over 400 °C).

High pad areas (1 × 1 mm) were used in order to facilitate electrical connection of the sensor structures with the package terminals [6,11,12].

Packaging is an essential process for temperature sensors operating at hundreds of degrees, in harsh environments. Several capsule models were tested for 1000 cycles of 50 °C–400 °C sweeps, with a variation rate of 50 °C/min. The temperature limits and swing speed correspond to general industrial equipment conditions. Following these tests, the TO39 package was selected, due to its good performances when working in harsh environments, specifically the hermetic sealing and good conductivity provided by its gold plated die pad and terminals. Figure 2b presents the bonding of a SiC-Schottky diode chip to the TO39 terminals. The anode is contacted through an Au wire with 25 µm thickness, while the cathode adheres directly to the die pad. For the latter connection, a few attachment techniques were investigated, using an intermediary layer (high temperature metal alloy preforms or nano-particle silver paste) [10,11], a solid-state diffusion (Au–Au between chip and die-pad) [28] and a transient liquid-phase bonding [29,30].

Using an intermediary binding layer proved to be most effective, with either nano-particle silver paste (Figure 2b) or preform [6,7,8,10,11,13,14]. For operating temperatures up to 400 °C, the only available preform was Au-In. Bonding using this solution is cumbersome because the process requires a high sintering temperature and a reduced ambient atmosphere (forming gas or H_2_, in order to prevent preform oxidation).

Die connections with nano-particle paste intermediary layer were obtained similarly with those resulting from a solid-state diffusion [25], that is, by applying pressure and heat for a specific duration. When using the silver paste, the nano-particles significantly increase contact pressure, which reduces the need for elevated temperature levels as well as the time demanded. Figure 2b shows an exemplification of cathode-die bonding using nano-particle silver paste.

The SiC-Schottky diode sensors, thusly packaged, were subjected to further thermal stress at 400 °C for 700 min. No damage was observed for either anode or cathode connections [10].

### 2.2. SiC-Schottky Diode Characterization

Forward low-bias behavior of ideal Schottky diodes is conventionally characterized by the thermionic emission (TE) equation, [6,7,8,9,10,11,12,13,31,32,33,34,35,36,37,38],

(1) $$I_{\mathrm{SD}}≅A_{n}A_{S}T^{2}\exp\left(-\frac{\Phi_{\mathrm{Bn},T}}{V_{\mathrm{th}}}\right)\exp\left(\frac{V_{\mathrm{SD}}}{nV_{\mathrm{th}}}\right).$$

In Equation (1), A_n_ is the Richardson constant (equal to 146 A/K^2^cm^2^ for 4H-SiC [6]), A_S_ is the Schottky contact area, T is the absolute temperature and V_th_ is its associated thermal voltage. The series resistance effect was not accounted for in the equation. The conventional barrier height (Φ_Bn,T_) and ideality factor (*n*) are critical device parameters, whose values and stability are closely linked with the technological process. They can be extracted, at each investigated temperature, from the slope and intercept of linear ln(I_SD_) as a function of V_SD_ plots (see Equation (1)) [6,7,8,9,10,11,12,13,14].

While, ideally, Φ_Bn,T_ and *n* should be temperature-independent, value variation for these parameters has been ubiquitously observed for Schottky diodes [6,7,8,9,10,11,12,13,14,31,32,33,34,35,36,37,38]. This phenomenon is attributed to inhomogeneities present on the Schottky contact surface, which leads to spatial variations of the Schottky barrier height (SBH). This topic was widely investigated, with many propositions of characterization methodologies [6,7,8,13,31,32,33,34,35,36,37,38]. One such recent development directed towards high-temperature (room to 450 °C) behavior modeling was proposed for Ni/SiC Schottky diodes [7,8,13]. The model considers a linear dependence of the conventional Schottky barrier [8]:

(2) $$\Phi_{\mathrm{Bn},T}≅\Phi_{\mathrm{Bn},\mathrm{eff}}+p_{eff}V_{\mathrm{th}},$$

where Φ_Bn,eff_ is an effective barrier height which can be temperature-stable over large spans [8,13]. The model also employs a non-uniformity parameter (*p_eff_*), which gives the temperature dependence of the conventional Schottky barrier [8,13].

Considering Equation (2), the current expression can be rewritten as [8,13]

(3) $$I_{\mathrm{SD}}≅A_{n}A_{S}T^{2}\exp\left(-p_{eff}-\frac{\Phi_{\mathrm{Bn},\mathrm{eff}}}{V_{\mathrm{th}}}\right)\exp\left(\frac{V_{\mathrm{SD}}}{nV_{\mathrm{th}}}\right).$$

Hence, the effective barrier and non-uniformity parameter can be determined from the $\ln\left(\frac{I_{\mathrm{SD}}}{A_{n}A_{S}T^{2}}\right)$ versus $\frac{T_{0}}{T}$ Arrhenius plot. T_0_ is a reference temperature, usually 300 K. A high *p_eff_* value usually indicates poor contact quality and significant mismatch between expected and actual current levels through the diode as temperature changes.

When the diode is under constant current bias, its forward voltage has a quasi-linear temperature dependence [13] (derived from Equation (3)):

(4) $$V_{\mathrm{SD}}\left(T\right)={n\Phi }_{\mathrm{Bn},\mathrm{eff}}-\left[{n\Phi }_{\mathrm{Bn},\mathrm{eff}}+2{\mathrm{nV}}_{\mathrm{th}0}\ln\left(\frac{T}{T_{0}}\right)-V_{\mathrm{SD}}\left(T_{0}\right)\right]\frac{T}{T_{0}},$$

where V_th0_ is the thermal voltage at the reference temperature T_0_. Equation (4) is essential for evincing a Schottky diode’s performances as a temperature sensor. It can be seen that sensitivity (the slope of V_SD_ in respect to T) is directly proportional to the values for the ideality factor and effective barrier height [6,7,8,9,10,11,12,13]. Because Φ_Bn,eff_ decreases with *p_eff_* (Equation (2)), it results that diode sensitivity is inversely proportional with the non-uniformity parameter. Moreover, adequate sensor operation can only be guaranteed for the temperature intervals where *n* and Φ_Bn,eff_ are constant, as any variations will lead to sensitivity fluctuations and, hence, a degradation in linearity [34,35].

### 2.3. Sensor Read-Out

The output signal of SiC-Schottky diodes used as temperature sensors is the forward voltage. To reconcile this aspect with the industrial requirements for sensors (current-mode response in the 4 mA–20 mA range), an acquisition and conversion circuit was developed.

**Figure 3:**
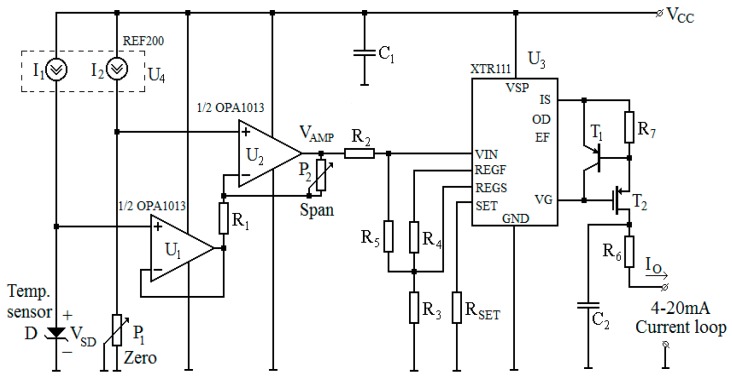
Electrical schematic of the read-out circuit [6].

The schematic of the proposed sensor read-out circuit is given in Figure 3 [12]. Two identical current generators (I_1_, I_2_—Figure 3) are necessary: one for forward biasing the SiC-Schottky sensor and the other for injecting constant current into a potentiometer (P_1_—Figure 3) [12,39]. P_1_ is used to set the minimum temperature detection threshold (usually 0 °C). This value corresponds to a read-out circuit output of 4 mA. A high stability, integrated double current source, REF200 [40], was used to perform these functions. Thus, any current variations owing to temperature or process fluctuations are similar for both injected currents.

The diode and potentiometer voltages were acquired by an instrumentation amplifier and implemented with the dual OPA1013 precision amplifier [41] (Figure 3). An important property of this amplifier is that the output can swing near ground despite using a single supply, which greatly reduces PCB area, complexity, and costs. A second potentiometer (P_2_—Figure 3) is used for tuning the gain of this amplifier. Essentially, it controls the span of the read-out circuit. Thus, the output voltage of the amplifier (V_AMP_) will swing between 0 V and 5 V as temperature varies between the minimum and maximum limits [12,39]. The V_AMP_ dependence in respect to sensor output signal (V_SD_) is determined from the schematic (Figure 3):

(5) $$V_{\mathrm{AMP}}=I_{2}\cdot R_{P_{1}}\cdot \left(1+\frac{R_{P_{2}}}{R_{1}}\right)-V_{\mathrm{SD}}\cdot \frac{R_{P_{2}}}{R_{1}}.$$

Equation (5) demonstrates that an iterative tuning of P_1_ and P_2_ can lock the amplifier’s full output swing (0 V–5 V) to a specific, desired temperature detection range.

The translation to the industrial standard current range is achieved using the XTR111 precision circuit [42], which is especially suited to current-loop transfer. Resistors R_2_–R_5_ are used together with the internal reference voltage V_R_ for scaling V_AMP_ to the nominal input range of the converter. The level shift is necessary so that when V_AMP_ is 0 V, the output current of the converter becomes 4 mA. The voltage-current conversion slope is adjusted through a single component, resistor R_SET_, leading to an overall tunable trans-conductance gain. Under these conditions, the output current of the read-out circuit is [42]:

(6) $$I_{o}=\frac{m}{R_{\mathrm{SET}}\cdot \left(R_{2}+R_{5}\right)}\cdot \left[R_{2}\cdot V_{R}\cdot \left(1+\frac{R_{4}}{R_{3}}\right)+R_{5}\cdot V_{\mathrm{AMP}}\right].$$

Equation (6) was determined taking into account the internal 1:m (with m = 10) precision current mirror of the XTR111 [42]. Thus, from Equations (5) and (6), a linear dependence between the sensor signal and read-out circuit output current is achieved. This makes for an overall linear temperature-current conversion, which is a major advantage over consecrated solutions based on thermocouples, where the Seebeck coefficient has a non-linear temperature variation [17].

**Figure 4:**
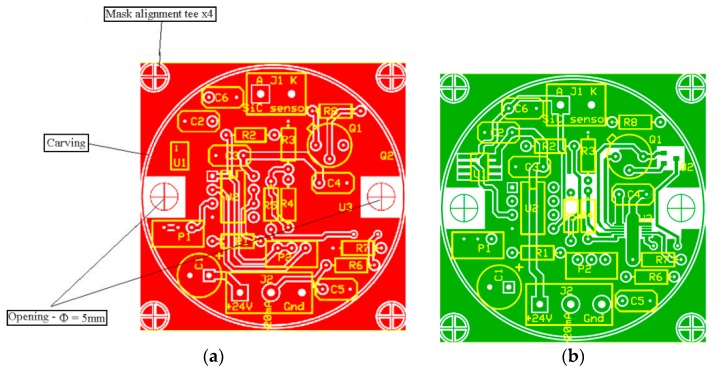
Layout of the read-out circuit: (**a**) Frontside and (**b**) Backside.

The layout for the read-out circuit (Figure 3) was designed, as shown in Figure 4, and implemented on a double-layered board (Figure 4a,b, respectively). The ground plane was used in order to minimize small-signal noise.

## 3. Results and Discussion

### 3.1. Schottky Diode Sensing Performances

Forward characteristics of fabricated SiC-Schottky diodes were measured up to 400 °C and parameterized in order to identify temperature-sensing performances, especially in regard to sensitivity and linearity. Measurements were performed using a Keithley 4200 Semiconductor Characterization System (Tektronix/Keithley, Cleveland, OH, USA) [6,7,8,9,10,11,12,13]. Temperature was controlled by means of a Varian oven, with a 1 °C resolution [43]. The main parameters (Φ_Bn,T_, Φ_Bn,eff_, *n*, *p_eff_*—see Equations (1) and (2)) were extracted for all investigated devices [6,7,8].

**Figure 5:**
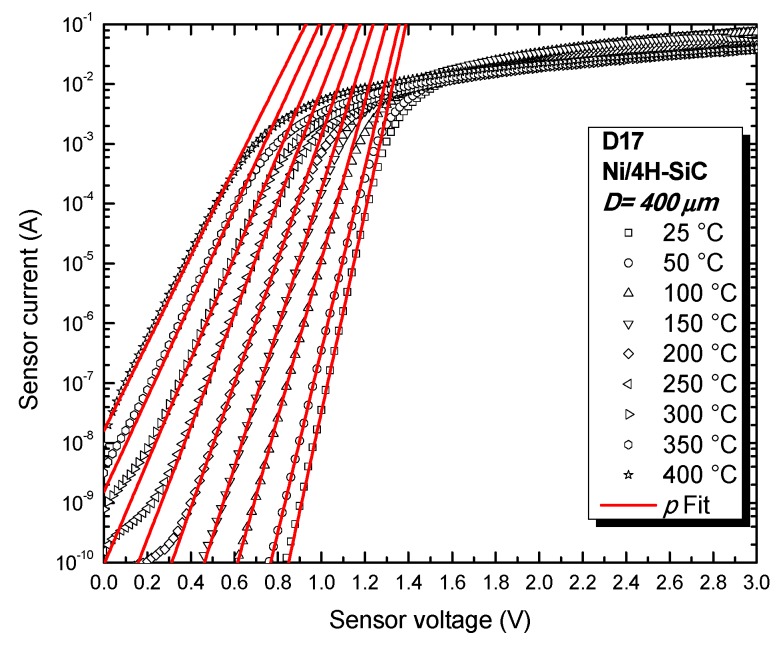
Forward characteristics and fitted curves for diode D17.

**Figure 6:**
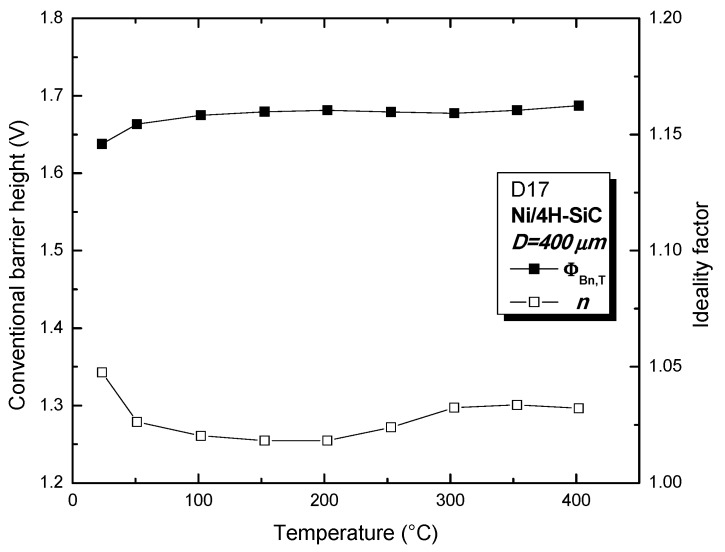
Conventional barrier height and ideality factor as a function of temperature.

Figure 5 depicts I_SD_-V_SD_ experimental curves, selected for one of the best performing samples (D17). It can be seen that the diode exhibits exponential current-voltage behavior over at least five orders of magnitude, even at 400 °C. The excellent device properties were also confirmed by the nearly temperature-independent values for the conventional barrier height (Φ_Bn,T_ ≅ 1.68 V, typical for annealed Ni/4H-SiC Schottky contacts [8]) and ideality factor (*n* ≅ 1.03, very close to unity), given in Figure 6. The effective barrier height and non-uniformity parameter are Φ_Bn,eff_ ≅ 1.64 V and *p_eff_* = 1.05, indicating only a slight degree of contact inhomogeneity [7,8,13]. These values were used in Equation (3) to fit the exponential portion of D17’s forward characteristics, as shown in Figure 5 (red lines). A very good agreement can be observed between experimental data and analytical results. Outstanding fitting accuracy was identified at the 100 nA–1 mA interval, indicating that D17 can operate adequately as a temperature sensor when biased in that current range for the entire 25 °C–400 °C domain.

**Figure 7:**
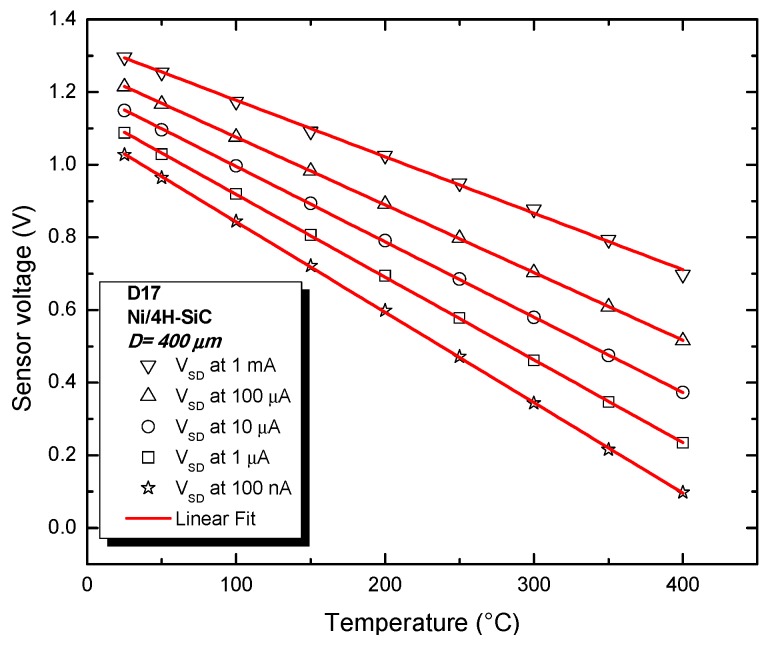
Sensor voltage as a function of temperature, at several bias currents: measurements (symbols) and fitting (red lines).

**Figure 8:**
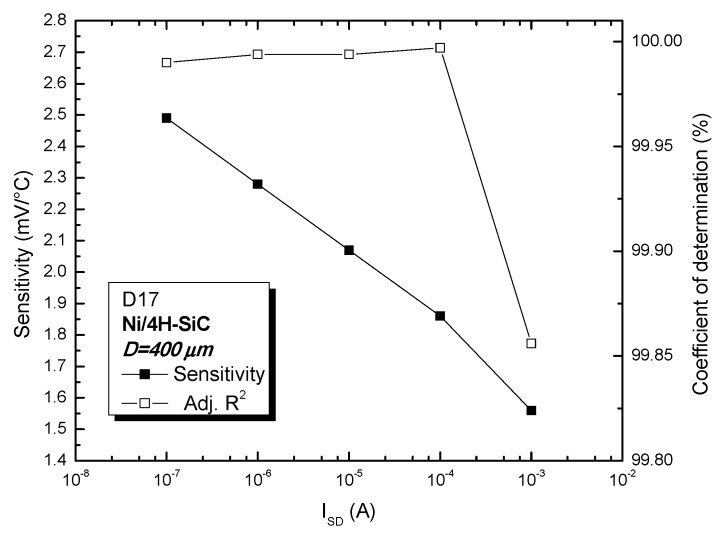
D17 sensing performances: sensitivity and linearity at various bias currents.

The practical temperature-sensing performances of D17 were determined by plotting the sensor voltage temperature dependence for various currents and linear fitting, as illustrated in Figure 7. The linearity, evinced by the coefficient of determination (adjusted R^2^), is shown in Figure 8, alongside sensitivity level (S—slope of fitted line). As expected, the elevated Schottky barrier of the device yields high values for sensitivity (over 10 times greater than thermocouples). Sensor linearity also slightly increases with bias current up to a current of 100 µA (Figure 8). Above this value, the impact of the parasitic series resistance becomes significant, leading to the small linearity decay at 1 mA. It can be noted that R^2^ values are greater than 99.99% for sensor currents over three orders of magnitude (Figure 8), proving that associated sensitivity values are strongly invariable over the entire 25 °C–400 °C range. The best value for R^2^ (99.997%—see Figure 8) was a key criterion for selecting I_SD_ = 100 µA for the proper operation of the temperature sensor. The lower sensitivity associated with this bias point can be compensated by the read-out circuit’s tunable gain.

**Figure 9:**
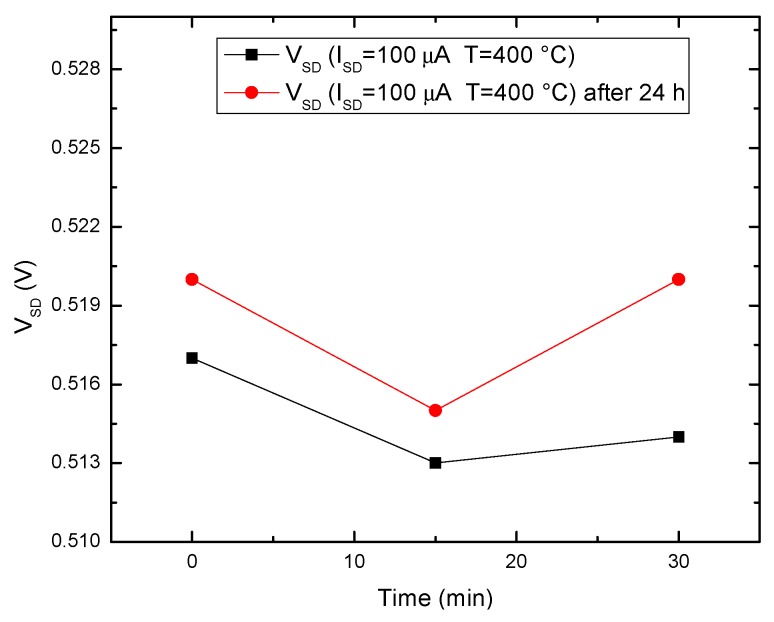
Sensor voltage stability at 400 °C and 100 µA bias.

The diode’s output signal stability at this constant current with temperature was also assessed [14]. The device was subjected to multiple cycles of 25 °C–400 °C swings for 24 h. Figure 9 depicts the initial and final V_SD_ values, measured at 400 °C over a 30 min interval. Overall variations are under 4 mV only, leading to an error around 0.5%.

### 3.2. Read-Out Circuit Calibration

After the practical implementation of the read-out circuit (Figure 3 and Figure 4), its electrical parameters were adjusted in order to obtain a linear response in the measurement interval of interest (25 °C–400 °C).

Circuit pre-calibration was achieved by inputting the corresponding sensor voltages (V_SD_) for 25 °C and 400 °C (obtained from the forward characteristics in Figure 5). These voltages were emulated with a multi-turn potentiometer. Afterwards, P_1_ (Figure 3) was adjusted in order to set the circuit’s output bottom threshold, 4 mA (corresponding to V_SD_ ≅ 1250 mV at ~25 °C—Figure 5). P_2_ sets the span of the output and was tuned in order to obtain a current of 20 mA for approximately 500 mV (corresponding to the sensor diode’s forward voltage at ~400 °C—Figure 5).

**Figure 10:**
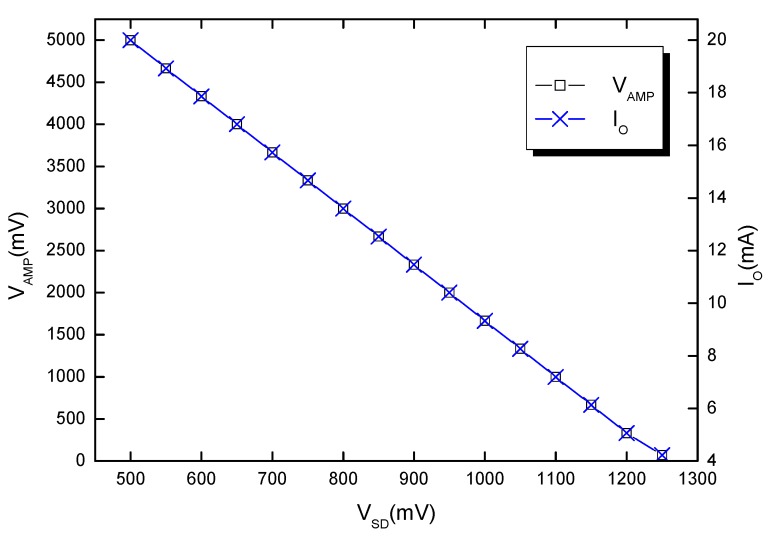
Voltage and current transfer characteristics of the read-out circuit.

Figure 10 shows the pre-calibration results for the read-out circuit, catered to D17 data (Figure 5). An identical dependence of V_AMP_ (output voltage of the instrumentation amplifier—Figure 3) and read-out circuit current (I_O_—Figure 3) as a function of sensor signal can be observed. The plots also evince very good linearity for the input range of interest. The slight discrepancies in the 1200 mV–1250 mV interval (Figure 10), associated with low measured temperatures (under 40 °C), are caused by the single-sided supply of the amplifier. This has little impact on the practical applications of the sensor-probe, as the monitored industrial processes usually take place at significantly higher temperatures.

The response time of the read-out circuit was tested by applying a ramp signal at its input by means of a programmable signal generator [12]. Thus, a voltage pulse, with a rise time of tens of ns, was generated and used as a substitute for V_SD_. The read-out circuit’s output shifted between 4 mA and 20 mA at a rate of approximately 5 mA/µs, which corresponds to an equivalent temperature swing of 117 °C/µs. This value greatly exceeds the actual rate of change for most industrial processes.

### 3.3. Industrial SiC-Schottky Temperature Probe

As mentioned before, Industrial temperature-monitoring probes usually consist of a sensing element and processing circuitry, packaged in a metal or ceramic casing (a protecting tube with connection head), with lengths varying in the 20 mm–2 m range.

**Figure 11:**
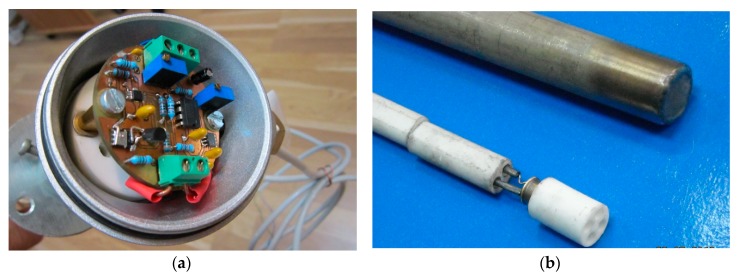
Integration into the industrial probe casing of: (**a**) Read-out circuit, placed in the connecting head and (**b**) SiC-Schottky diode sensor, fitted at the end of the protecting tube.

The PCB layout of the read-out circuit (Figure 4) was therefore especially designed to be mounted inside the connecting head of a standard industrial temperature measurement casing, as displayed in Figure 11a. The Schottky diode-sensor’s packaging allowed for placement at the end of the probe’s protecting tube (Figure 11b). The connection wires crossing through the tube were electrically insulated using ceramic coating, as also evinced by Figure 11b.

Thus, using the best performing sensor-diodes, several fully electrically and mechanically compatible industrial temperature probes were developed, with the following characteristics:

- Supply voltage: +24 V;
- Supply insulation from the grounded probe casing;
- Standard industrial current mode output in the 4 mA–20 mA range;
- Power consumption under 1.2 W;
- Ability to work in harsh conditions (dust, humidity, vibrations, high temperatures) for an extended period of time.

**Figure 12:**
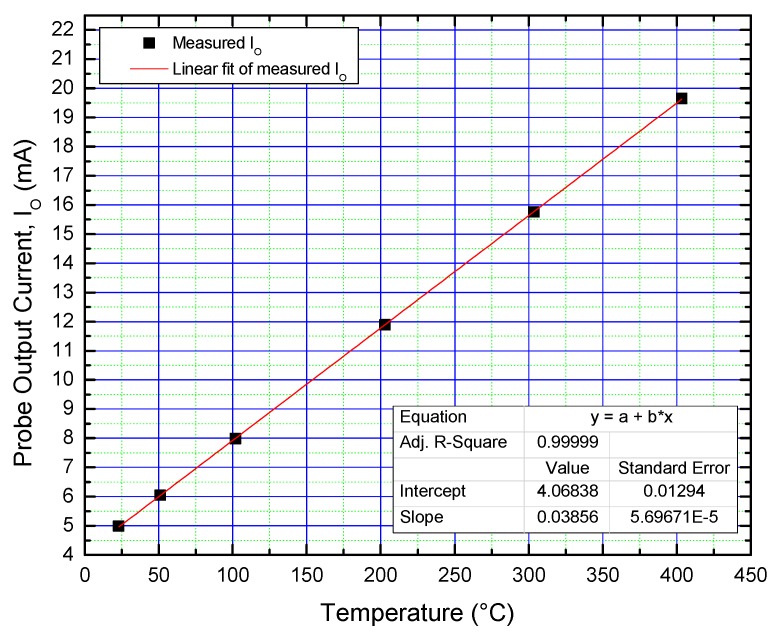
Probe output current as a function of temperature (with D17 as sensing element).

The industrial sensors were calibrated inside the Varian oven between 25 °C and 400 °C, yielding their current-temperature conversion characteristics. One such curve is depicted in Figure 12, for the probe using D17 as a sensing element. It can be observed that, even considering the low-temperature linearity decay, the adjusted R^2^ is very close to ideality (99.999%—Figure 12). Probe sensitivity was determined from the slope of the transfer characteristic, at 38.5 µA/°C (Figure 12).

**Figure 13:**
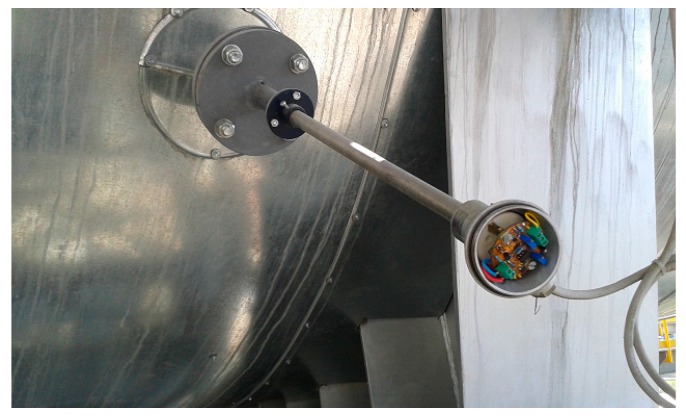
Industrial SiC-Schottky temperature probe mounted at the output of the raw meal mill. Connecting head open in order to reveal read-out circuit mechanical compatibility. D17 as sensing element.

After the calibration process, the probes were integrated into the sensor network of the cement factory at Fieni [44], in order to monitor key points in the fabrication process. The factory has seven clinker kilns, eight paste mills, a raw meal mill, ten cement mills, two lime kilns and a section for production of prefabricated asbestos cement parts. The SiC-Schottky diode-based probes were mounted, alongside conventional thermocouple probes, at the input (high-temperature) and output (low-temperature) of the raw meal mill. The former incorporation is illustrated in Figure 13.

**Figure 14:**
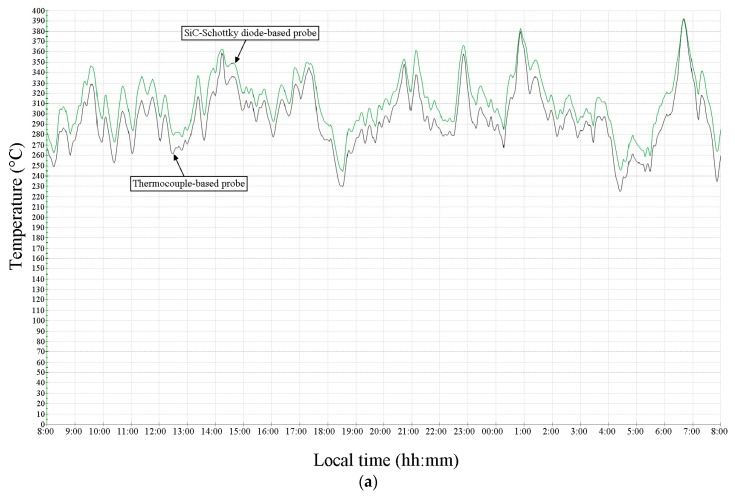

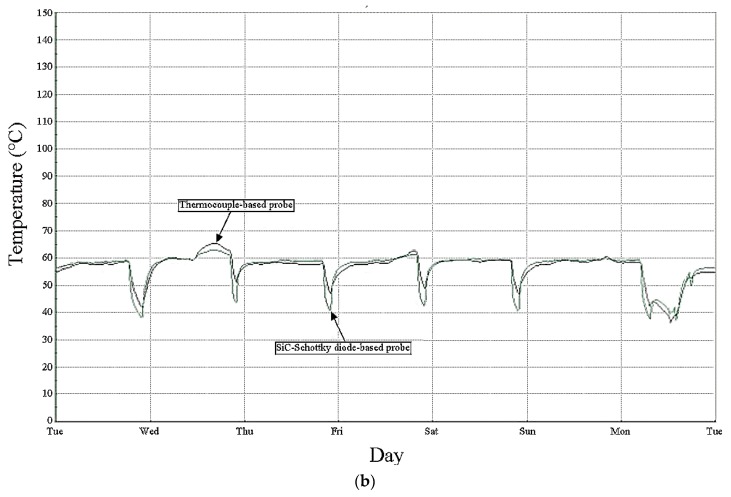
Temperature measurement results for SiC-Schottky temperature probe (green) and thermocouple-based probe (black) at (**a**) the input and (**b**) the output of the raw meal mill from Fieni cement factory.

The output signals of both temperature sensor pairs (SiC-Schottky and thermocouple) were monitored simultaneously in the control room of the factory. The temperature waveforms are shown in Figure 14, measured at the mill input (Figure 14a) and its output (Figure 14b).

The results presented in Figure 14 show very good agreement between SiC-diode and thermocouple-based sensors, over very wide temperature ranges, and up to 400°C. The slight variations (around 5%) can be attributed to the fact that the two compared sensors were not located in the same casing and could not be thermally coupled. The SiC-sensors were placed approximately 2 m ahead of conventional thermocouple-based probes. Naturally, the discrepancies are more pronounced for measurements at the raw meal mill output (Figure 14a) because of the higher operation temperatures. The longest lifetime for a SiC-Schottky diode-based probe during these industrial tests was around 6 months, which is about two times longer than conventional solutions.

**Figure 15:**
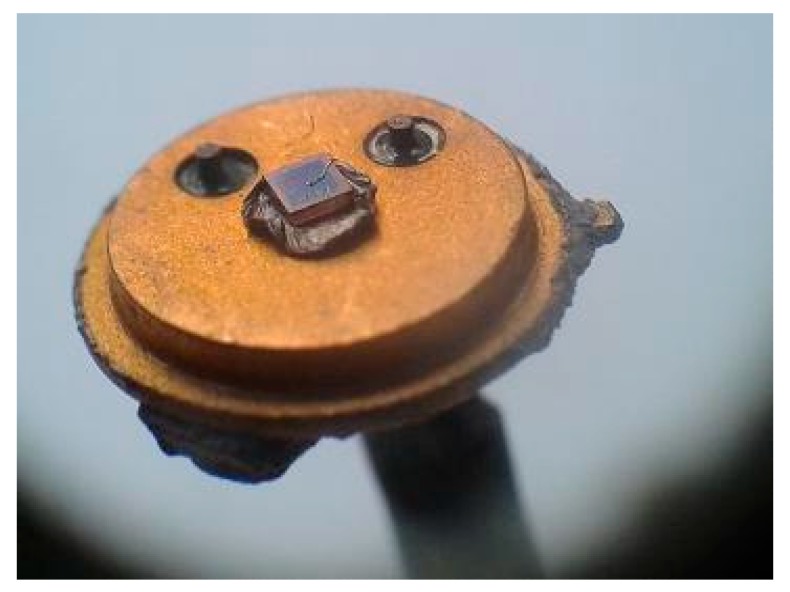
Schottky sensor structure with broken Au wire, after operation in real industrial conditions. Die-pad bonding with Au-In preform (comparison with Figure 2b—bonded with silver nano-paste).

The most common fault of the proposed temperature probe was caused by the breaking of the Au wire connecting the SiC-Schottky diode’s anode to the TO39 terminal, as exemplified in Figure 15.

## 4. Conclusions

A high-temperature probe with SiC Schottky diodes as sensing element was developed for operating in the cement industry. Full electrical and mechanical compatibility with existing factory sensors was proven.

4H-SiC devices with annealed Ni/4H-SiC Schottky contacts were fabricated, packaged, measured up to 400 °C, and fully parameterized in order to assess temperature-sensing properties and identify best performing devices. Exemplary diode temperature sensitivities were between 1.8 mV/°C and 2.54 mV/°C, for bias currents in the range 100 nA–100 µA. Excellent sensing linearity (with R^2^ higher than 99.99%) was also exhibited in this current interval.

A dedicated read-out circuit was developed so as to convert the sensor output signal to the industrial standard requirements. The circuit architecture and subsequent PCB design were dedicatedly attuned to factory conditions regarding supply voltage (single-ended, 24 V), probe output (current-mode response with range of 4 mA–20 mA) and physical size (matching the connecting head of an industrial probe casing). A high response time (117 °C/µs) for the processing circuit was achieved, much greater than the temperature variation speed in the operation environment.

High-performance SiC diode-sensors and associated read-out circuits were incorporated in standard industrial casings, and the ensembles were tested inside a cement factory. High and low-temperature points, in a raw meal mill, were selected for monitoring, alongside thermocouple-based counterparts. Very close agreement was identified between proposed and conventional temperature sensing solutions, between 40 °C and 400 °C. The best probe lifetime was 6 months.

These promising results evince the capability of SiC device-based sensors to operate in real harsh industrial conditions and constitute an important milestone for silicon carbide applications. Future developments include the automation of the calibration process and lowering the bias current of the SiC-Schottky sensor (through minimal read-out circuit reconfiguration), in order to increase sensitivity. This is possible because the casing acts as an electro-magnetic shield, greatly reducing external noise.
